# Cloning and expression of two new prolactin-related proteins, prolactin-related protein-VIII and -IX, in bovine placenta

**DOI:** 10.1186/1477-7827-3-68

**Published:** 2005-12-07

**Authors:** Koichi Ushizawa, Toru Takahashi, Misa Hosoe, Kanako Kaneyama, Kazuyoshi Hashizume

**Affiliations:** 1 Reproductive Biology and Technology Laboratory, Developmental Biology Department, National Institute of Agrobiological Sciences, 2 Ikenodai, Tsukuba, Ibaraki 305-8602, Japan; 2 Department of Veterinary Medicine, Faculty of Agriculture, Iwate University, 3-18-8 Ueda, Morioka, Iwate 020-8550, Japan; 3 Department of Technology, National Livestock Breeding Center, 1 Odakurahara, Odakura, Nishigo, Fukushima 961-8511, Japan

## Abstract

**Background:**

Prolactin-related proteins (PRPs) are specific proteins of the growth hormone/prolactin (GH/PRL) family in bovine placenta. This study reports the identification and sequencing of a full-length cDNA for two new members of bovine PRPs, bPRP-VIII and -IX, and their localization and quantitative expression in bovine placenta.

**Methods:**

New bPRP-VIII and -IX were identified from bovine placentome. Localization and quantitative gene expression in the placenta were respectively investigated by *in situ *hybridization and real-time RT-PCR methods. Recombinant proteins of these genes were produced by a mammalian HEK293 cell expression system.

**Results:**

Full-length bPRP-VIII and -IX cDNA were respectively cloned with 909 and 910 nucleotide open-reading-frames corresponding to proteins of 236 and 238 amino acids. The predicted bPRP-VIII amino acid sequence shared about 40 to 70% homology with other bPRPs, and bPRP-IX had about 50 to 80 % homology of others. The two new bPRPs were detected only in the placenta by RT-PCR. mRNA was primarily expressed in the cotyledon and intercotyledonary tissues throughout gestation. An *in situ *hybridization analysis revealed the presence of bPRP-VIII and -IX mRNA in the trophoblastic binucleate and/or trinucleate cells. bPRP-VIII mRNA was observed in the extra-embryonic membrane on Day 27 of gestation, however, no bPRP-IX mRNA was observed in the extra-embryonic membrane in the same stage of pregnancy by quantitative real-time RT-PCR analysis. Both new bPRP genes were possible to translate a mature protein in a mammalian cell expression system with approximately 28 kDa in bPRP-VIII and 38 kDa in bPRP-IX.

**Conclusion:**

We identified the new members of bovine prolactin-related protein, bPRP-VIII and -IX. Localization and quantitative expression were confirmed in bovine placenta by *in situ *hybridization or real-time PCR. Their different temporal and spatial expressions suggest a different role for these genes in bovine placenta during gestation.

## Background

Prolactin-related proteins (PRPs) are members of the growth hormone/prolactin (GH/PRL) family [1]. PRPs are highly expressed in binucleate cells of bovine trophoblasts, although their function is still obscure. No new member had been identified between early 1990 and this year [2]. We recently found a new *PRP *member through a comprehensive analysis using a bovine utero-placental cDNA microarray [3]. Seven bovine *PRP *(*bPRP*) genes have been identified in placentomal tissues, whereas only two of those genes have been shown to be translated. The expression pattern of these genes is spatially and temporally different. *bPRP-I *indicates the considerable importance of regulation of implantation and formation of the placentome in bovines [4,5]. However, *bPRP-VII *was expressed in intercotyledon and cotyledon with rather weak compare to those of *bPRP-I *during the gestation [6].

Placental lactogens (PLs) are known as classical members and PRPs are categorized as the non-classical members of the GH/PRL family in bovine [7]. Various non-classical members are common in rodents, such as mice and rats, and some of them exhibit known biological functions, such as proliferin and prolactin-like protein-A (PLP-A) [7-12]. However, as a whole the biological function of Prolactin-related genes is still not known in various species including bovine. Two research groups identified bovine *PRPs *mRNA individually; *bPRP-I *to -*III *were identified by the Schuler group [13,14], and *bPRP-IV *to -*VI *were identified by the Nakashima group [15-17]. We recently identified another one named as *bPRP-VII *[6]. Our previous study suggested our bovine placental cDNA library contained other *bPRP *genes [3]. We reveal here the full-length sequences of two new members of *bPRPs, bPRP-VIII and -IX*, and their localization and quantitative expression. We confirmed the possibility of translation of them and the accuracy of these gene sequences to produce a recombinant protein using the HEK293 cell-transfecting system.

## Materials and methods

### Animals and tissues

Placental tissues for cDNA cloning and mRNA expression were collected from Japanese Black cows. The extra-embryonic tissues, placenta, and endometrium were collected at a local slaughterhouse on days 27 to 28, 56 to 64, 144 to 149, and 245 to 258 after artificial insemination (day 1). The tissues were separated into four portions: the cotyledon (COT); intercotyledon (the area between the cotyledonary villous (ICOT)); the caruncle area, including the maternal placentomal septa in the endometrium (CAR); and the intercaruncle area (ICAR). It was difficult to divide the COT and ICOT on days 27 to 28, and thus the COT contained very few villi. Tissues from two different cows on day 27 and one cow on day 28 of gestation (n = 3) were used as Day 27 extra-embryonic membrane (Day 27EEM), Day 27 caruncle (Day 27CAR), and Day 27 intercaruncular endometrium (Day 27ICAR). Placentomal tissues were collected on days 56, 58, and 64 (in total, n = 3) and designated as Day 60COT, CAR, ICOT, and ICAR. Sample materials from days 144, 148, and 149 (n = 3) and days 245 (two samples) and 252 (one sample) were marked as Day 150COT, CAR, ICOT, ICAR, Day 250COT, CAR, ICOT, and ICAR. The cotyledonary and caruncular part was separated mechanically, and each part may contained a counter part of tissue. The collected samples were stored at -80°C until RNA extraction. The placentomes of day 60 were fixed in 3.7% formaldehyde PBS at pH 7.4 and then embedded in paraffin wax and stored at 4°C until *in situ *hybridization. All procedures for these animal experiments were carried out in accordance with the guidelines and ethics approved by the Animal Ethics Committee of the National Institute of Agrobiological Sciences for the use of animals.

### Cloning of full-length bPRP-VIII and -IX cDNA

The new full-length *bPRP-VIII and -IX *cDNA were isolated from bovine cotyledonary tissue by the 3'-rapid amplification of cDNA ends (RACE) method. In brief, a complete RNA was isolated from a bovine placentome on day 60 of gestation using ISOGEN (Nippon Gene, Toyama, Japan). We performed 3'-RACE using a 3'-full RACE core set (Takara, Kyoto, Japan) with *bPRP-VIII*-specific forward primer (5'-CCACAGTCAACAGGAGTCCTCA-3') and *bPRP-IX*-specific forward primer (5'-CCAACAGAGAGTCCTCACCCTGCGA-3'). The *bPRP-VIII and -IX *primer was designed from bovine EST accession number AW464912 and BP108069, respectively. The 3'-RACE products were sequenced using an ABI Prism 370 automatic sequencer (Applied Biosystems, Foster City, CA, USA) after cloning into a pGEM-T Easy vector (Promega, Madison, WI, USA).

### Phylogenetic analysis

Alignments of deduced protein sequences were performed with the multiple alignment software Clustal W 1.83 on the DDBJ web site. Clustal W was also employed to calculate trees using the Neighbor-Joining (NJ) method [18]. TreeView was used to display the phylogenetic tree [19]. The values represent bootstrap scores for 1,000 trials, indicating the credibility of each branch. Except the bPRP-VIII and -IX sequences, all the bPRPs and bPLs protein sequences were obtained from GenBank. Their GenBank accession numbers are: bPRP-I (J02944), bPRP-II (M27239), bPRP-III (M27240), bPRP-IV (M33269), bPRP-V (AB239755), bPRP-VI (X59504), bPRP-VII (AB187564), bPL-Ala (J02840), and bPL-Val (M33268).

### Three-dimensional structure prediction by FAMS

We predicted the three-dimensional (3D) structure of bPRP-VIII and -IX by using the FAMS (Fully automated homology modeling system; ) [20]. FAMS is the software which predicts 3D model of the target protein from the structural known protein of high homology. In case of bPRP-VIII and -IX, the 3D structure was constructed based on the human prolactin (hPRL) 3D structure (Protein Data Bank ID: 1N9D) in the element. FAMS program only requires an amino acids sequence as input, and constructs 3D model structures automatically. Visualization of the 3D structure was performed using the RasMol 2.7.3 software [21].

### RT-PCR

**Figure 1:**
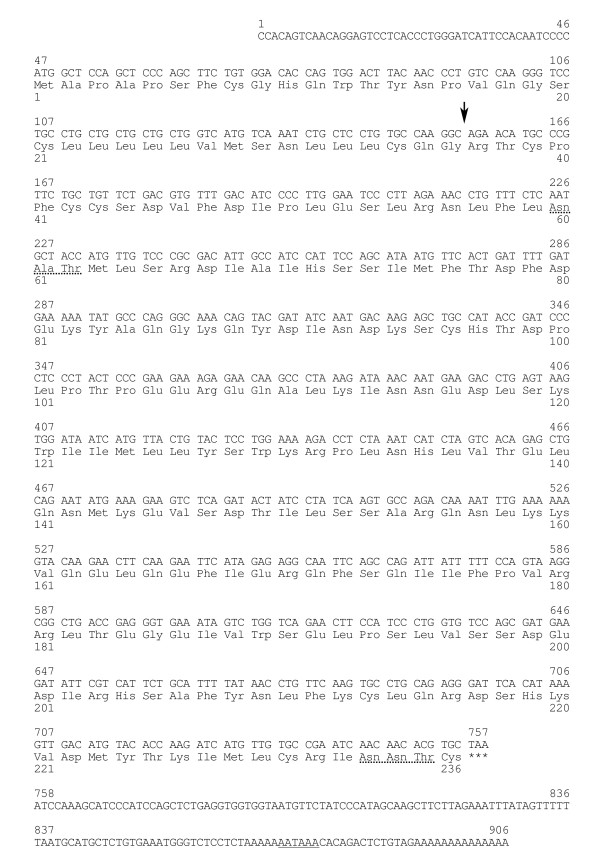
**Nucleotide and deduced amino acid sequences of *bPRP-VIII***. The arrow indicates the putative primary cleavage site of the signal peptide. The potential *N*-glycosylation site is underlined with a dotted line. The asterisks indicate the termination codon. The polyadenylation signal is underlined with a solid line.

Tissue distribution of *bPRP-VIII and -IX *expression was studied by RT-PCR. Bovine GAPDH was used as a positive control for the PCR. Details of the RT-PCR method were described in previous reports [6,22]. The total RNA in a total reaction mixture was used for reverse transcription and template cDNA synthesis using oligo(dT) primer and Superscript II reverse transcriptase (Invitrogen, Carlsbad, CA, USA) at 42°C for 50 min. Each PCR contained the cDNA template, primers, deoxynucleotide triphosphate mixture (dNTP), MgCl_2_, 10 × PCR buffer II, autoclaved milliQ water, and AmpliTaq gold DNA polymerase (Applied Biosystems). Amplification conditions included denaturation at 95°C for 30 sec and extension at 72°C for 1 min. Twenty-seven cycles were performed for all samples. The annealing temperature was set at 60°C for 30 sec. A single denaturation step at 95°C for 10 min before the first PCR cycle and a final extension step at 72°C for 10 min after the last PCR cycle were also performed. The PCR products were analyzed by agarose gel electrophoresis and visualized by ethidium bromide staining. The primers encoding for the *bPRP-VIII and -IX *sequences were designed using the sequence illustrated in Fig. 1. The primer sequence was selected to include a mismatch for other bPRPs. The designated primers are listed in Table 1. All the primers were commercially synthesized (Espec Oligo Service, Tsukuba, Japan).

**Table 1:** Oligonucleotide primers used for RT-PCR

**Gene**	**Primer**	**Sequence**	**Position**
*bPRP-VIII*	Forward	5'-AGAACATGCCCGTTCTGCTGT-3'	155–175
(AB196438)	Reverse	5'-TTAGCACGTGTTGTTGATTCG-3'	757-737
*bPRP-IX*	Forward	5'-AACTCATGCCCATCCTGTGGT-3'	150–170
(AB204881)	Reverse	5'-TTAGCACTTTTTGCGGATTCG-3'	758-738
*GAPDH*	Forward	5'-CCTTCATTGACCTTCACTACATGGTCTA-3'	71–98
(U85042)	Reverse	5'-GCTGTAGCCAAATTCATTGTCGTACCA-3'	927-901

### *In situ *hybridization

Full-length cDNA of *bPRP-VIII and -IX *was used as a template for hybridization probe synthesis. Digoxigenin (DIG)-labeled antisense and sense-complementary RNA probes were prepared as described in previous studies [6,23]. The placentomes were sectioned into 7 μm-thick sections for hybridization. *In situ *hybridization was performed using automated Ventana HX System Discovery with a RiboMapKit and BlueMapKit (Ventana, Tucson, AZ, USA) [6]. Briefly, sections were hybridized with DIG-labeled probes in RiboHybe (Ventana) hybridization solution at 65°C for 6 hours. The sections were washed three times in RiboWash (Ventana) (65°C, 6 min) after hybridization and were fixed in RiboFix (Ventana) (37°C, 10 min). The hybridization signals were then detected with monoclonal-anti-digoxin biotin conjugate (Sigma, Saint Louis, MI, USA). The hybridized glasses were observed after preparation with a Nikon ECLIPSE E800 photomicroscope (Nikon, Tokyo, Japan).

### Real-time RT-PCR

Gene expression of *bPRP-VIII and -IX *was confirmed quantitatively at each stage of gestation (Days 27FM, 27CAR, 27ICAR, 60COT, 60CAR, 60ICOT, 60ICAR, 150COT, 150CAR, 150ICOT, 150ICAR, 250COT, 250CAR, 250ICOT, and 250ICAR) by real-time RT-PCR analysis. Details of the real-time RT-PCR procedures have been described in previous reports [6,24]. Briefly, fifty ng of the total RNA were reverse transcribed into cDNA for 30 min at 48°C by MultiScribe™ reverse transcriptase with an Oligo dT primer, dNTP mixture, MgCl_2 _and RNase inhibitor. Primer pairs and oligonucleotide probes labeled with a reporter fluorescent dye at the 5'-end and a quencher fluorescent dye at the 3'-end were designed using the Primer Express computer software program (Applied Biosystems). The primers and probes for each gene are listed in Table 2. The thermal-cycling conditions included initial sample incubation at 50°C for 2 min and at 95°C for 10 min, followed by 40 cycles at 95°C for 15 sec and at 60°C for 1 min. The cycle threshold values (C_T_) indicate the quantity of the target gene in each sample and were determined in real time using an ABI Prism 7700 sequence detector (Applied Biosystems). The relative difference in the initial amount of each mRNA species (or cDNA) was determined by comparing the C_T _values. The standard curves for each gene were generated by serial dilution of plasmid containing *bPRP-VIII*, -*IX*, or *GAPDH *cDNA to quantify the mRNA concentrations. The ratio of *bPRP-VIII and -IX *mRNA to *GAPDH *mRNA was calculated to adjust for any variations in the RT-PCR reaction. All values are presented as mean ± SD. Statistical analysis was performed using one-way ANOVA followed by the Tukey-Kramer multiple comparison test. Differences were considered significant at *P *< 0.05.

**Table 2:** Oligonucleotide primers and TaqMan probes used for real-time RT-PCR analysis

**Gene**	**Primer or TaqMan probe**	**Sequence**	**Position**
*bPRP-VIII*	Forward	5'-CAAGGGTCCTGCCTGCTG-3'	98–115
(AB196438)	Reverse	5'-GGCATGTTCTGCCTTGGC-3'	164–344
	Probe	5'-TGCTGCTGGTCATGTCAAATCTGCTCC-3'	117–143
*bPRP-IX*	Forward	5'-ATATGCCCAGGGCAAACTGT-3'	287–306
(AB204881)	Reverse	5'-TCGGGAGCATGGAAGGAAT-3'	358-340
	Probe	5'-TATCAATGCCACCAACAGCTGCCACA-3'	311–336
*GAPDH*	Forward	5'-AAGGCCATCACCATCTTCCA-3'	178–197
(U85042)	Reverse	5'-CCACTACATACTCAGCACCAGCAT-3'	253-230
	Probe	5'-AGCGAGATCCTGCCAACATCAAGTGG-3'	200–225

TaqMan probes of bPRP-VIII and -IX have 5'-FAM dye and 3'-TAMRA dye

TaqMan probe of GAPDH has 5'-VIC dye and 3'-TAMRA dye

### Production of recombinant proteins

The* bPRP-I*, *-VIII*, *-IX*, and *bPL* sequences encoding the mature protein region, which combined the FLAG and 6 × His epitope tag sequences, were inserted into the pFLAG-CMV-3 vector (Sigma). The constructed plasmid was transfected into HEK 293 cells using FuGENE 6 (Roche Diagnostics, Basel, Switzerland) for transient transfection. Stably transfected HEK 293 cells were adapted to the suspension culture in a spinner flask using 293 SFM II medium (Invitrogen, Gibco) and cultured in an atmosphere of 5% CO_2 _in air at 37°C for 3 days. The medium was separated by centrifugation and stored at -30°C.

### Western blot analysis

The 10 μg of proteins from the HEK293 cell conditioned media were loaded on each lane, separated by SDS-PAGE, and electrophoretically transferred onto a polyvinylidene difluoride membrane [25]. Western blotting was performed by the method of Towbin et al. [26]. Briefly, the membrane was blocked in 10% skim milk overnight, incubated with anti-FLAG M2 (Sigma) for 1 h at room temperature, followed by incubation with anti-mouse IgG conjugated with alkaline phosphatase (Sigma) (diluted 1:3000) for 1 h at room temperature. Immunopositive bands were stained using NBT (Bio-Rad, Hercules, CA, USA) and BCIP (Bio-Rad).

## Results

### Sequences of bPRP-VIII and -IX cDNA

**Figure 2:**
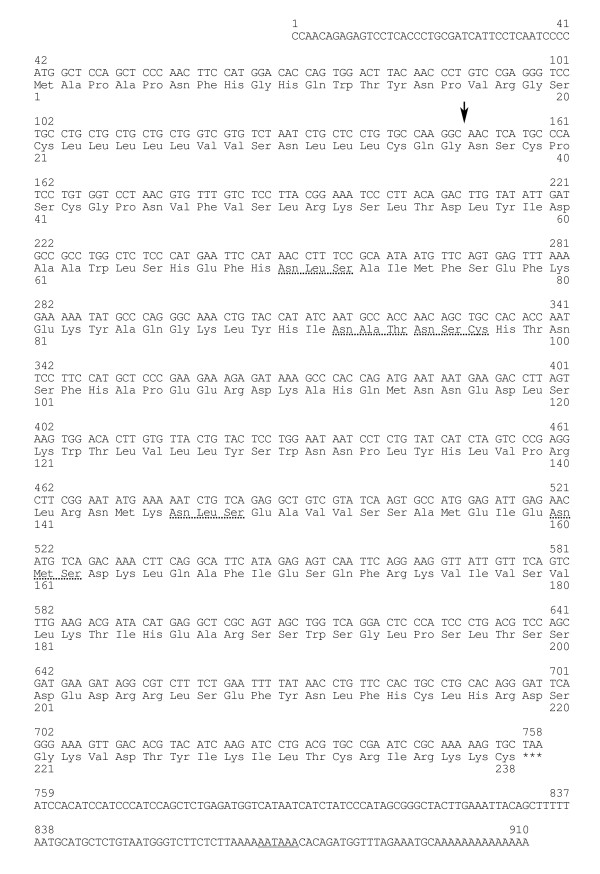
**Nucleotide and deduced amino acid sequences of *bPRP-IX***. The arrow indicates the putative primary cleavage site of the signal peptide. The potential *N*-glycosylation site is underlined with a dotted line. The asterisks indicate the termination codon. The polyadenylation signal is underlined with a solid line.

**Figure 3:**
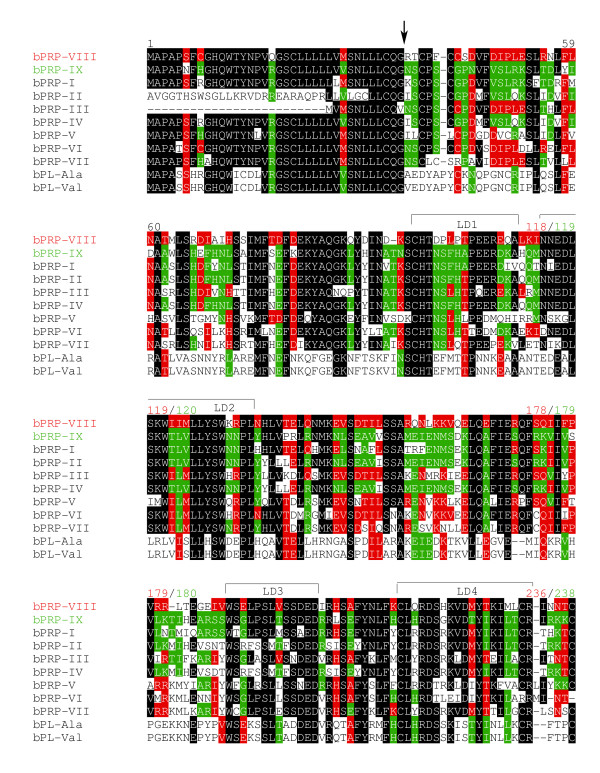
**Comparison of amino acid sequences of bPRP-VIII and -IX with other members of bPRPs and bPLs**. Residue present in both bPRP-VIII and -IX is shown in black boxes. Residue present in only bPRP-VIII is designated in red boxes. Residue present in only bPRP-IX is designated in green boxes. Amino acid sequences were aligned with assistance from Clustal W 1.83 on the DDBJ web site. The arrow indicates the putative primary cleavage site of the signal peptide of bPRP-VII. LD1, 2, 3, and 4 refer to the conserved domain in the prolactin family.

**Figure 4:**
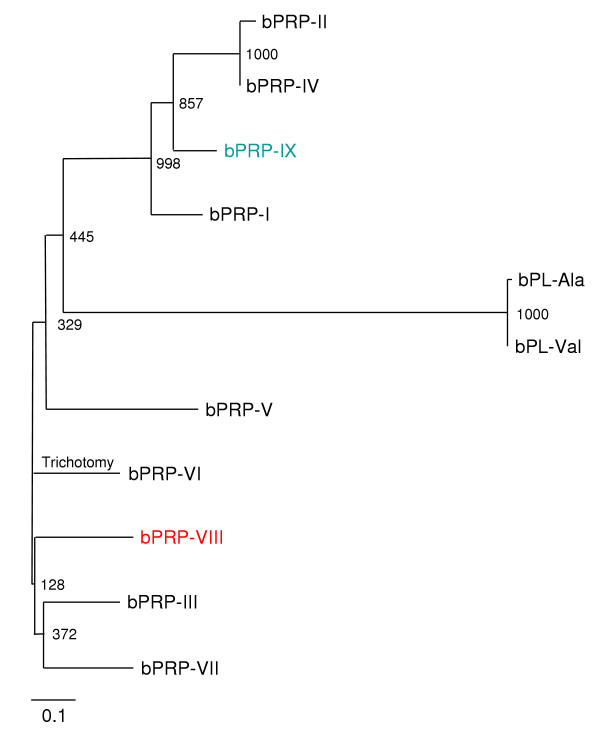
**Phylogenetic tree of bPRPs and bPLs**. The tree was constructed using TreeView following alignment of the protein sequences by the Clustal W 1.83 algorithm. The numbers at the base of each branch division represent bootstrap values after 1000 repeats. Scale bar represents 0.1 amino acid replacements per amino acid site. For GenBank/DDBJ accession numbers, refer to Materials and Methods.

**Figure 5:**
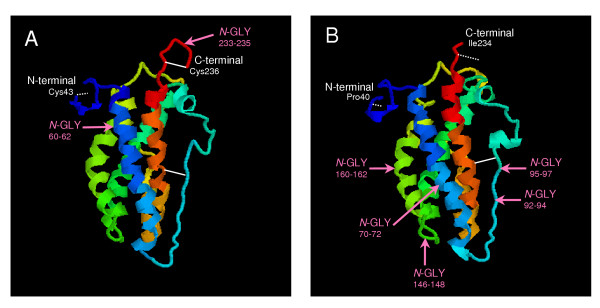
**The predicted 3D structure of (A) bPRP-VIII and (B) bPRP-IX mature protein**. The 3D structure were predicted by FAMS software. bPRP-VIII structure was able to construct from Cys43 to Cys236 amino acid region. bPRP-IX structure was able to construct from Pro40 to Ile234 amino acid region. Disulfide bonds refer to white solid line. Predicted disulfide bonds refer to white dot line. *N*-GLY refer to potential *N*-glycosylation site.

Full-length *bPRP-VIII and -IX *were cloned from bovine placentome. The 906- and 910-nucleotide sequences were isolated in *bPRP-VIII and -IX*, respectively (Fig. 1 and 2). The protein sequence regions (CDSs) were composed of 711 nucleotides in *bPRP-VIII *and 717 nucleotides in *bPRP-IX*. The 3'-untranslated region contains one AATAAA polyadenylation signal start 20 and 26 bases upstream from the poly (A) addition site in *bPRP-VIII and -IX*, respectively. The amino acid sequences deduced from full-length *bPRP-VIII *and *bPRP-IX *cDNA are amino acids 236 and 238. The homology of predicted amino acid sequences of bPRP-VIII and -IX protein were shown in Fig. 3. The predicted sequence of bPRP-VIII protein was 69% homologous to that of bPRP-VI, 66% homologous to that of bPRP-VII, 61% homologous to that of bPRP-I and -III, 58% homologous to that of bPRP-IV and -V, 57% homologous to that of bPRP-IX, 42% homologous to that of bPRP-II, and 39% homologous to that of bPL-Ala (Fig. 3). The predicted sequence of bPRP-IX protein was 81% homologous to that of bPRP-IV, 76% homologous to that of bPRP-I, 70% homologous to that of bPRP-II, 60% homologous to that of bPRP-VII, 57% homologous to that of bPRP-VI and -VIII, 53% homologous to that of bPRP-III and -V, and 40% homologous to that of bPL-Ala (Fig. 3). In the phylogenetic analysis, it was shown that bPRP-VIII was close to bPRP-III, bPRP-VI, and bPRP-VII sides in the phylogenetic tree and bPRP-IX was close to bPRP-II and bPRP-IV sides in the phylogenetic tree (Fig. 4). The *N*-terminal regions of the bPRP-VIII and -IX proteins were rich in hydrophobic amino acid residue, which is characteristic of the signal peptide. bPRP-VIII had two consensus sequences for *N*-glycosylation and Asn-X-Ser/Thr at the positions of 60 to 62 and 233 to 235 (Fig. 1). bPRP-IX also had four consensus sequences for *N*-glycosylation at the positions of 70 to 72, 92 to 94, 146 to 148, and 160 to 162 (Fig. 2). Another atypical *N*-glycosylation site, Asn-X-Cys was found in only bPRP-IX at the position of 95 to 97, and this region is identified in bPLs. The TAA stop codon was used in both bPRP-VIII and -IX, and appeared after the sequence TGC, which was present in other bPRPs except for bPRP-VI and bPLs that encode C-terminal cysteine residue [17]. The predicted 3D structures of bPRP-VIII and -IX mature region are shown in Fig. 5. The structural differences of *N*-glycosylation site, disulfide bond (-S-S-) and each atomic configuration were confirmed. We submitted these sequences to the DNA Data Bank of Japan (DDBJ). The DDBJ/GenBank accession Nos. are AB196438 and AB204881.

### Expression of bPRP-VIII and -IX mRNA in bovine placenta

**Figure 6:**
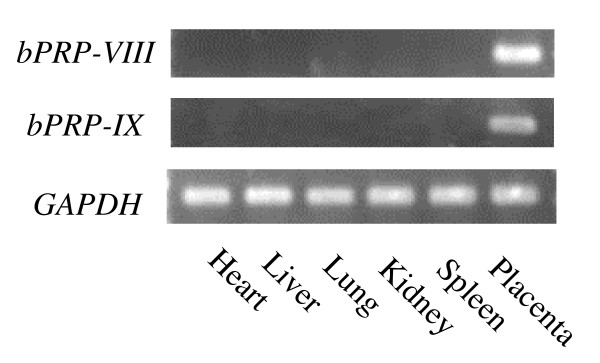
**Expression of *bPRP-VIII and -IX *mRNA in bovine tissues**. Heart, liver, lung, kidney, and spleen were used by RT-PCR. Cotyledonary tissue at day 148 of gestation was used as a placental sample. *GAPDH *expression in each tissue is presented as standard data.

The distribution of *bPRP-VIII and -IX *mRNA was examined in various bovine tissues by RT-PCR. Both *bPRP *mRNAs appeared only in the placental tissue (Fig. 6).

**Figure 7:**
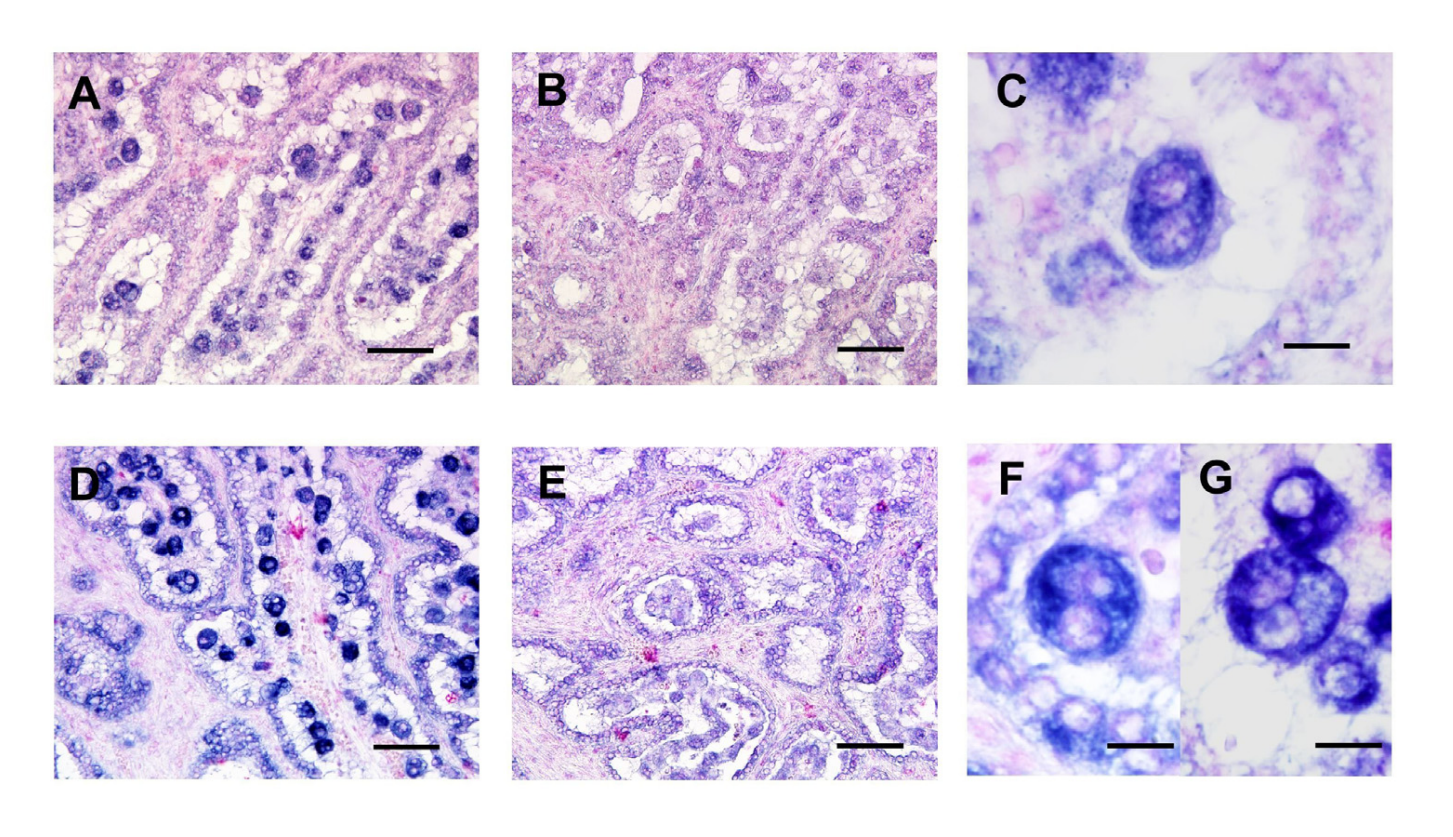
**Localization of *bPRP-VIII and -IX *in the bovine placentome on day 60 of gestation. **(A, B, C) *bPRP-VIII *and (D, E, F, G) *bPRP-IX *mRNA were detected by *in situ *hybridization. (A, C, D, F, G) DIG-labeled anti-sense cRNA probes were used. (B, E) DIG-labeled sense cRNA probes were used. Seven-microgram sections of bovine placentome were hybridized with each probes. Scale bar = 100 μm on the (A, B, D, E) and 20 μm on the (C, F, G).

*bPRP-VIII and -IX *mRNA localization was determined by *in situ *hybridization in the bovine placentome on day 60 of gestation (Fig. 7). DIG-labeled *bPRP-VIII and -IX *anti-sense RNA probes specifically detected the mRNA transcript in the placentome and intercotyledonary membrane. Both *bPRPs *appeared in the binucleate cells in the cotyledon and intercotyledonary membrane, and *bPRP-IX *mRNA was also detected in trinucleate cells (Figs. 7D and 7F). No significant signals were detected with sense probes in any genes (Fig. 7B and 7E).

**Figure 8:**
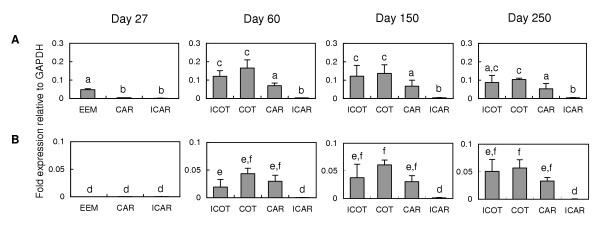
**Quantitative real-time RT-PCR analysis of (A) *bPRP-VIII *and (B) *bPRP-IX *mRNA in bovine placenta**. The total RNA was extracted from cotyledons containing extra-embryonic membrane (EEM), cotyledonary placenta (COT), intercotyledonary fetal membrane (ICOT), caruncular placenta (CAR), and intercaruncular endometrium (ICAR) on Day 27, Day 60, Day 150, and Day 250 of gestation. The expressions of these mRNAs were normalized to the expression of *GAPDH *measured in the same RNA preparation. Values are means ± SD. Values with different letters are significantly different (*P *< 0.05).

Quantitative real-time RT-PCR analysis indicated that *bPRP-VIII *mRNA appeared primarily in the extra-embryonic membrane of Day 27, cotyledon and intercotyledonary membrane of Day 60 to 250 (Fig. 8). The expression intensity increased approximately four-fold in the cotyledon from Day 27 to Day 60, and then slightly decreased until Day 250 (approximately 0.6-fold). The expression intensity increased approximately three-fold in the intercotyledon from Day 27 to Day 60 and was maintained from Day 60 to Day 150. Then it then decreased slightly until Day 250 (approximately 0.7-fold). In the caruncular area, the expression increased by about four to five times by Day 60, and the expression level was maintained to late gestation. However no significant expression was detected in the intercaruncle. *bPRP-IX *mRNA was also detected in the cotyledonary placentome and intercotyledonary membrane after Day 60 of gestation, but it did not appear in the extra-embryonic membrane on Day 27 of gestation. The mRNA expression intensity increased approximately 1.2-fold from Day 60 to Day 150 in the cotyledon and approximately 1.9-fold the intercotyledon, and then those values were maintained until Day 250. No expression of *bPRP-IX *was observed in the caruncular area on Day 27, but stable expression intensities were detected from Day 60 to Day 250. These expression intensities were rather strong compared to the intercotyledonary and cotyledonary areas, even though the sources for this gene may be binucleate and trinucleate cells.

**Figure 9:**
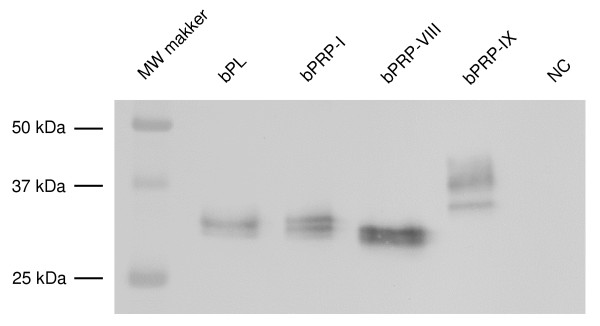
**Production of recombinant bPRP-VIII, bPRP-IX, bPRP-I, and bPL proteins**. Conditioned media from HEK 293 cells transiently transfected with each gene were collected, and the proteins (10 μg) were loaded in separate lanes. The proteins were separated by SDS PAGE and specific proteins were detected by Western blot analysis using an anti-FLAG tag. MW Marker: molecular weight marker. NC: Negative control (transfected vector only).

The recombinant proteins of bPRP-VIII and -IX raised with HEK293 cell were detected by Western blot analysis (Fig. 9). The intense bands of bPRP-VIII with a FLAG epitope tag migrated to approximately 28 kDa molecular weight, while those of bPRP-IX with a FLAG epitope tag migrated to 38 kDa, 34 kDa and 26 kDa (Fig. 9).

## Discussion

In the present study, we identified two new members of *bPRP *cDNAs, *bPRP-VIII and -IX*. Totally nine *bPRP *genes have been identified at this moment in bovine.

*bPRP-VIII and -IX *cDNA sequences had following features, respectively. The position of the polyadenylation signal (AATAAA) in the *bPRP-VIII *started 20 bases upstream from the poly(A) additional site. This configuration is specific to *bPRP-VIII*. The number of bases in *bPRP-VIII *from the polyadenylation signal to poly(A) site are the fewest in known *bPRPs*. The position of the polyadenylation signal in the *bPRP-IX *started 26 bases upstream from the poly(A) additional site. This position coincides with those in *bPRP-II *and *bPRP-IV *[14,15]. The signal peptide sequences in the *N*-terminal regions of both bPRP-VIII and -IX were conserved similarly to those in other bPRPs/bPLs (Fig. 3). The signal cleavage site was predicted to be between the Glu-36 and Arg-37 in bPRP-VIII, and between Glu-36 and Asn-37 in bPRP-IX by homology to the *N*-terminal regions of the bPRPs/bPLs (Fig. 3). As a result, bPRP-VIII is predicted to be a mature protein composed of 200 amino acids, while bPRP-IX has 202 amino acids. bPRP-VIII lacked two amino acids (positions 94 and 183), which is a sequence characteristic, since other known bPRPs have 202 amino acids. bPRP-VIII had two potential *N*-glycosylation sites (amino acid portions 60 to 62 and 233 to 235, Figs. 1, 3 and 5). The position of 60 to 62 coincided with those of bPRP-III, -VI, and -VII [6,14,17], however, the second *N*-glycosylation site was found at 233 to 235 in amino acids. In contrast, bPRP-IX had four potential typical *N*-glycosylation sites of Asn-X-Ser/Thr (amino acid portions 70 to 72, 92 to 94, 146 to 148, and 160 to 162, Figs. 2, 3 and 5). All these configurations of Asn-X-Ser/Thr coincide with those of bPRP-II and -IV [14,15]. However, it is characteristic that only the bPRP-IX have atypical *N*-glycosylation site of Asn-X-Cys (amino acid portions 95 to 97). The conserved domains (LD1 to LD4, Fig. 3) were suggested by Yamakawa et al. [15] in other prolactin-related genes. The bPRP-VIII and -IX sequences also revealed these LD domains. Cysteine residues (PRP-VIII positions 96, 213, and 230, and PRP-IX positions 97, 215, and 232) in the LD1 and LD4 domains were conserved in bPRPs/bPLs, except for bPRP-VI. bPRP-VIII had four more cysteines (positions 38, 41, 42, and 236, Fig. 3, a total of seven residues in mature protein), and bPRP-IX had three more cysteines (positions 38, 41, and 238, Fig. 3, a total of six residues in mature protein). These configurations indicate common features in bPRPs/bPLs. In particular, the configuration of the cysteine residue in bPRP-VIII coincided exactly with that of bPRP-III in the mature sequence region. The cysteine residue in bPRP-IX was also the same as that in bPRP-I, -II, and -IV in the mature sequence region. Therefore, both bPRP-VIII and -IX may have three disulfide bonds similar to those of other bPRPs, except for bPRP-VII [6].

The primary mRNA expression of *bPRP-VIII and -IX *was observed in the binucleate cells (Fig. 7). Both genes could independently produce mature recombinant proteins in the mammalian cell expression system (Fig. 9). The binucleate cells may have produced the bPRP-VIII and -IX proteins simultaneously because *bPRP-VIII and -IX *mRNA expressed in the binucleate cells, and HEK293 cells translated these mRNA to each protein individually. Binucleate cells are also primary expression cells for *bPRP-I *and *bPL *[27,28] and may have a specific function for implantation in the fetomaternal interface [11,29]. *bPRP-VIII *mRNA was also expressed in the extra-embryonic membrane just after the implantation period in the present study (Day 27 of gestation) (Fig. 8). bPRP-VIII may also be related to the implantation process like bPRP-I and -VII since *bPRP-I *was expressed during early gestation. Compare to these expression, no *bPRP-IX *was present in the extra-embryonic membrane on Day 27 of gestation (Fig. 8). This mRNA expression pattern implies that bPRP-IX has a different role from *bPRP-I *and *bPL *in placental formation [6]. *bPRP-IX *mRNA was also detected in the trinucleate cells. A trinucleate cell is generated by migration of a binucleate cell to an endometrial epithelial cell with the progress of the pregnancy [30]. bPRP-IX may be a necessary molecule during middle to late gestation. Although both bPRP-VIII and -IX may be necessary molecules for gestation, *in situ *hybridization demonstrated that both genes were primarily expressed in binucleate and trinucleate cells. This means both genes derive from fetal side origin, but both were stably contained in the caruncular area throughout gestation, except bPRP-IX in early gestation. There are two potential causes of this result. One is contamination after separation. A second possibility is cell migration [30]. The present study and our previous study suggest that binucleate and trinucleate cells may migrate deeply into the endometrium after implantation and maintain their functions, which may be related to immunomodulation or stress during bovine gestation [6,7,30,31]. The molecular size of the recombinant proteins completely differed between bPRP-VIII and -IX. Transcripted bPRP-VIII protein is of relatively smaller size in the known bPRP family members, and the bPRP-IX protein is among the largest members [6,32,33]. The sugar chain addition in post-translational modification will give large effect in the difference of both molecular weights. It was found that the predicted 3D structure differed clearly in both molecules, because configuration of disulfide bonds and sugar chain addition dynamics are different. The tertiary structure of bPRP-VIII and -IX are similar to that of hPRL, so they may share the same receptor. However there is no information and the other evidence may not support this speculation. Only the *bPRP-I *gene has been known to produce protein in the placenta, however, the protein bound to the alpha2-macroglobulin [32], so it might have a biological function in paracrine status. Since all other bPRPs have a similar characteristic, it is difficult to say bPRPs are hormones secreted into circulation from the placenta, such as bPRL and bPL. It was possible to predict a part of their functions from the molecular weight, 3D structure, and post-translational modification. bPRP-VIII and -IX proteins may have different functions, since their temporal and special expressions were different. Various difficulties remain for understanding PRP functions, however, many of the same genes and molecules are expressed in bovine placenta as in rodent placenta [7].

Recently, bovine genome projects have addressed various shotgun sequence data and five known *bPRPs*, *bPL*, and *bPRL*, a total of seven prolactin related genes, hit in the NCBI genome database. They are located in Bos taurus chromosomes 12 and/or 23. *bPRP-III*, -*VII *and *bPL *were detected on chromosome 23 with *bPRL*. On the other hand, *bPRP-I*, -*V*, and -*VI *are placed on chromosome 12. Rodents such as mice and rats also have various *PLPs *[7,34], and they have been clustered on one chromosome, number 13 in mice and number 17 in rats [34,35]. bPRP-III and -VII were in comparatively close positions on the phylogenetic tree in Fig. 4, and they are clustered in the same chromosome 23. *bPRP-VIII *may be placed on chromosome 23 because bPRP-VIII was close to bPRP-III or -VII on the phylogenetic tree, although the bootstrap value is low between bPRP-VIII and bPRP-III/-VII. bPRP-I, -V, and -VI, however, were comparatively close on the phylogenetic tree in Fig. 4; these genes were clustered on chromosome 12. *bPRP-IX *may be placed on chromosome 12 because bPRP-IX was close to bPRP-I on the phylogenetic tree. The actual configuration of the bPRP-VIII and -IX genome will be addressed in the future. Therefore, prolactin-related genes in mammals may have similar roles for placental function, but they may evolve through different phylogenetic processes, may be two separate pathways. For example, GH and PRL functions are different in various species, namely, GH uses the PRL receptor in some species, but not in others [7,36].

In conclusion, we identified two new members of *bPRPs, bPRP-VIII and -IX*. *bPRP-VIII *was expressed in binucleate cells in bovine trophoblast tissue and placentome. The expression appeared as well in *bPRP-I, -VII*, and *bPL*, and it expressed from the implantation period to late in gestation. *bPRP-IX *was expressed in binucleate cells and trinucleate cells, and was expressed somewhat late in gestation compared to other PRPs. These data indicate that various *PRP *genes in bovine placenta have coordination roles for gestation as evidenced in rodents.
